# Sex differences in middle cerebral artery reactivity and hemodynamics independent from changes in systemic arterial stiffness in Sprague–Dawley rats

**DOI:** 10.14814/phy2.70250

**Published:** 2025-04-01

**Authors:** Jonathan W. Ray, Xuming Sun, Nildris Cruz‐Diaz, Victor M. Pulgar, Liliya M. Yamaleyeva

**Affiliations:** ^1^ Department of Surgery/Hypertension and Vascular Research Center Wake Forest University School of Medicine Winston‐Salem North Carolina USA; ^2^ Department of Pharmaceutical and Clinical Sciences Campbell University Buies Creek North Carolina USA

**Keywords:** arterial stiffness, cerebral hemodynamics, cerebral reactivity, middle cerebral artery, sex differences, transcranial Doppler ultrasound

## Abstract

The mechanisms of sex differences in cerebrovascular function are not well understood. In this study, we determined whether sex differences in middle cerebral artery (MCA) reactivity are accompanied with changes in cerebral or systemic arterial resistance and stiffness in young adult Sprague–Dawley (SD) rats. No differences in systolic or diastolic blood pressures were observed between sexes. Heart rate was higher in the female versus male SD. Left MCA pulsatility index (PI) was lower in female versus male SD. No differences in left intracranial internal carotid artery (ICA) PI were observed between sexes. There were no differences in thoracic aorta or left common carotid artery pulse wave velocity (PWV) between sexes. In isolated MCA segments, female left MCA had lower contraction to potassium, but similar maximal contraction and sensitivity to thromboxane A2 receptor agonist U46619. Pre‐incubation with indomethacin lowered maximal response and sensitivity to U46619 in male but not female MCA. Endothelial nitric oxide synthase and vascular smooth muscle layer thromboxane A2 receptor immunoreactivity were greater in female versus male SD. We conclude that sex differences in the MCA reactivity are associated with a differential functional profile of MCA in adult SD rats independent from changes in systemic PWV.

## 1 INTRODUCTION

Cerebrovascular disease is a major risk factor for the development of cognitive impairment. It is now well understood that women are at greater risk for cerebrovascular disease (Bushnell et al., 2014). Women have a higher lifetime risk of stroke, a higher risk for Alzheimer's disease, and greater cognitive ability deficits in older age compared to men (Gao et al., 1998; Petrea et al., 2009; Read et al., 2006; Seshadri et al., 2006). Despite the debilitating effects of cerebrovascular disease in women, the basis for sex differences in cerebrovascular dysfunction remains incompletely understood. Furthermore, impaired vasodilation or cerebrovascular resistance in young or middle‐aged adults may indicate increased vulnerability for future risk of cerebrovascular disease.

The middle cerebral artery (MCA) is a major cerebral artery and the largest branch of the internal carotid artery (Navarro‐Orozco & Sanchez‐Manso, 2024). Among cerebral blood vessels, MCA and its branches are most commonly affected by stroke and therefore remain a focal point of research on cerebrovascular disease (Navarro‐Orozco & Sanchez‐Manso, 2024). Sex differences in the structure and function of the MCA are reported and likely contribute to the sex differences surrounding cerebrovascular disease severity and onset. Previous works show that female MCAs exhibit smaller internal diameter, less vascular smooth muscle cells (VSMCs), and increased collagen and elastin content as well as impaired cerebral blood flow autoregulation (Wang et al., 2020). Young women are relatively protected from cerebrovascular disease compared with older women or men; however, there is a rapid doubling of cerebrovascular disease in women in the decade following menopause (Bushnell et al., 2014; Lisabeth & Bushnell, 2012; Robison et al., 2019; Shekhar et al., 2017). The milieu of vasoactive sex hormones may contribute to sex differences in MCA dysfunction (Bushnell et al., 2014; Lisabeth & Bushnell, 2012; Robison et al., 2019; Shekhar et al., 2017). Indeed, sex hormones (including estrogens, progestins, and androgens) have been shown to exert influence over numerous functions of cerebral blood vessels including maintenance of cerebrovascular tone and cerebral blood flow, angiogenesis, vascular remodeling, inflammation, and maintenance of the blood–brain barrier (Robison et al., 2019). Furthermore, oral contraceptive use and hormone replacement therapy are sex‐specific risk factors for stroke in women (Bushnell et al., 2014; Girijala et al., 2017; Roy‐O'Reilly & McCullough, 2018). Additionally, greater peripheral arterial stiffness in women has been linked to increased pulsatile cerebral blood flow, a known contributor to the pathogenesis of cerebrovascular disease (Chung et al., 2017; Cruz‐Cosme et al., 2018; Lau et al., 2018; Lefferts et al., 2020; Mitchell et al., 2008; Purkayastha et al., 2014; Smulyan et al., 2001; Tarumi et al., 2014; Wahlin et al., 2014; Yang et al., 2016).

The early hemodynamic changes in MCA can be identified with noninvasive methods such as transcranial Doppler (TCD) ultrasound. TCD ultrasound analysis of the MCA provides valuable insight into cerebrovascular function in human subjects and laboratory animals (Giustetto et al., 2015; Kassab et al., 2007; Wielicka et al., 2020). Pulsatility index (PI), a measure of vascular resistance, is one of the parameters determined by the TCD ultrasound technique: increased PI indicates greater vascular resistance and potentially downstream hypoperfusion (Hussein et al., 2018). MCA PI has been also used to predict vascular cognitive impairment in hypertensive patients (Harris et al., 2018). In addition, the relationship between central pulse wave velocity and middle cerebral artery pulsatility and reactivity is not well understood and may depend on sex (Reeve et al., 2024). Since cerebrovascular function during early adulthood may provide insights into future brain health, in this study, we used young adult Sprague–Dawley rats to determine sex differences and the potential mechanisms underlying changes in MCA hemodynamics and systemic arterial stiffness.

## 2 MATERIALS AND METHODS

### 2.1 Animals

The study was approved by the Institutional Animal Care and Use Committee of the Wake Forest University School of Medicine (A21‐057). Young adult (25‐week‐old) male and female Sprague–Dawley (SD) rats were purchased from Charles River Laboratories (Wilmington, MA, USA). Rats were housed at a constant room temperature, humidity, and light cycle (12:12‐h light–dark), fed a standard rodent chow (Lab Diet 5P00 – Prolab RMH 3000, PMI Nutrition International, INC, Brentwood, MO) and given water ad libitum throughout the experimental protocols.

### 2.2 Blood pressure and heart rate measurements

Systolic (SBP) and diastolic (DBP) blood pressures and heart rate were recorded in trained, conscious rats by the tail‐cuff method using the Non‐Invasive Blood Pressure (NIBP) Monitor System (Columbus Instruments, Columbus, OH, USA). Data were averaged for each animal and reported as mean ± SD.

### 2.3 Transcranial Doppler ultrasound

**FIGURE 1:**
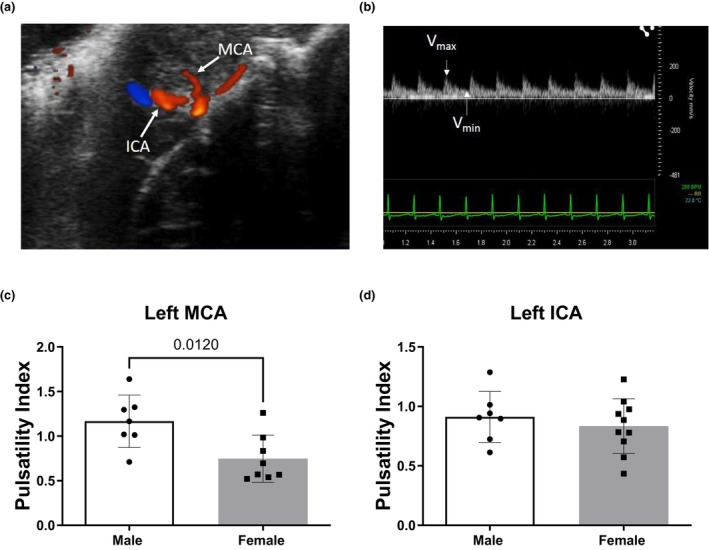
Transcranial Doppler ultrasound and pulsatility index (PI) of left middle cerebral artery (MCA) and left internal carotid artery in adult Sprague–Dawley (SD) rats. Color Doppler ultrasound was used to identify the LMCA which appeared as the first large branch arising from the intracranial internal carotid artery (Panel a). Waveforms obtained from pulse wave Doppler analysis provide maximum, minimum, and mean flow velocities which are used to calculate PI (Panel b). Left MCA (Panel c) and ICA (Panel d) PI in 25‐week‐old male versus female SD rats. Data are mean ± SD; *n* = 7–10.

High‐frequency ultrasound was used to determine the pulsatility index (PI) of the left MCA (LMCA) and intracranial portion of the left internal carotid artery (LICA). Animals were placed on a temperature‐controlled platform. Temporal hair was removed using a depilatory cream (Nair, Church & Dwight Co., Ewing, NJ). Ultrasound was performed using a Vevo LAZR Ultrasound and Photoacoustic System and LZ250 transducer (FujiFilm, VisualSonics, Toronto, Canada) under 1.5% isoflurane anesthesia. The LMCA is visualized in color Doppler mode by directing the transducer through the rat temporal foramen as previously described (Giustetto et al., 2015). Maximum (V_max_), minimum (V_min_), and mean (V_mean_) blood flow velocities were determined by pulse wave Doppler mode and were averaged over three cardiac cycles (Figure 1a,b). PI was calculated as follows: PI = V_max_‐V_min_/V_mean_. Data were analyzed using Vevo LAB software version 5.7.1 for Windows (FujiFilm VisualSonics, Toronto, Canada).

### 2.4 Pulse wave velocity measurements

Pulse wave velocity (PWV) of the thoracic aorta and left common carotid artery (LCCA) was determined as a measure of systemic vascular stiffness (Williams et al., 2007). The following methods of PWV calculations were used in aorta and left commons carotid artery: time from R of QRS complex to pulse wave Doppler impulse at the foot of proximal (t_1_) and distal points (t_2_), and the distance between t_1_ and t_2_ was used to calculate the PWV as follows: PWV = d/(t_2_–t_1_) as described by us (Elsangeedy et al., 2024).

### 2.5 Immunohistochemistry

LMCAs were fixed in 10% formalin and 70% ethanol, embedded in paraffin, and cut into 5‐μm sections. Immunostaining was performed using the avidin biotin complex (ABC) method with a diaminobenzene solution used as the chromogen. Antigen retrieval treatment with IHC‐TEK Epitope Retrieval Solution (IHC World, Woodstock, MD, USA; Cat #: IW‐1100) was applied at 95–98°C for 40 min. Nonspecific binding was blocked in a buffer containing 10% normal goat serum, 0.1% bovine serum albumin, and 1% Triton X‐100 in PBS for 30 min. LMCA sections were incubated with rabbit polyclonal cyclooxygenase 2 antibody (COX‐2; dilution 1:200; Cayman Chemical, Ann Arbor, MI, USA; Cat #: 160126), mouse monoclonal endothelial nitric oxide synthase antibody (eNOS; dilution 1:200; BD Biosciences, Franklin Lakes, NJ, USA; Cat #: 610297), rabbit polyclonal thromboxane A2 receptor antibody (TxA_2_R; dilution 1:1000; Alomone Labs, Jerusalem, Israel; Cat #: APR‐069), or rabbit polyclonal prostaglandin I synthase antibody (PGIS; 1:200 dilution; Cayman Chemical, Ann Arbor, MI, USA; Cat #: 100023) and secondary biotinylated goat anti‐rabbit or anti‐mouse antibodies (dilution 1:400; Vector Laboratories, Newark, CA, USA; Cat #: BA‐1000; Cat #: BA‐9200). Representative images were acquired from each slide with a Mantra Microscope at 40× magnification using Mantra Snap acquisition software (Perkin Elmer, Waltham, MA, USA). Regions of interest (ROI) were defined using the open‐source Fiji software (ImageJ, National Institutes of Health). COX‐2, TxA_2_R, and PGIS were analyzed in the endothelial and tunica media layers of MCA, while eNOS was analyzed in the endothelial layer of MCA. Intensity of the staining in five ROIs per segment was quantified as described previously by us following the reciprocal intensity method (Nguyen et al., 2013; Pulgar et al., 2014, 2015).

### 2.6 Vascular reactivity

LMCAs were dissected and mounted between an isometric force transducer (Kistler Morce DSC 6, Seattle, WA, USA) and a displacement device on a wire myograph (Multi Myograph, Model 620 M Danish Myo Technologies, Aarhus, Denmark) using two stainless steel wires (diameter 40 μm), using techniques previously described (Pulgar et al., 2014, 2015, 2019). The myograph organ bath (5 mL) was filled with KHB maintained at 37°C and aerated with 95% O_2_/5% CO_2_. The vessels were washed and incubated for 30 min before the normalization procedure was performed. Arterial segments were normalized to 0.9·L100, with L100 being the internal circumference the vessels would have if they were exposed to a transmural pressure of 100 mmHg. Each arterial segment was stretched in 50 μm steps, internal circumference (L) and wall tension at each stretch level were recorded to produce a resting wall tension‐internal circumference curve using the DMT Normalization Module (ADInstruments) (Pulgar et al., 2014, 2015, 2019). Optimal diameters (OD) were calculated as OD = 0.9·L100/π (Pulgar et al., 2014, 2015, 2019). Responses to agonists were recorded after an equilibration period of 30 min. *Response to acetylcholine*. LMCAs were washed and stimulated with a sub‐maximal dose of U‐46619 (10^−6.5^ M, Cayman Chemical, Ann Arbor, MI, USA; Cat #: 16450), once the contraction was stable a dose response curve to acetylcholine (10^−10^–10^−4^ M; Cayman Chemical, Ann Arbor, MI, USA; Cat #: 23829) was performed. *Response to the thromboxane analog U‐46619*. Contractile response to U‐46619 was tested on basal tone. LMCAs were exposed to 13 increasing concentrations of U‐46619 (10^−10^–10^−5^ M) applied in half‐long steps.

### 2.7 Statistical analysis

**FIGURE 2:**
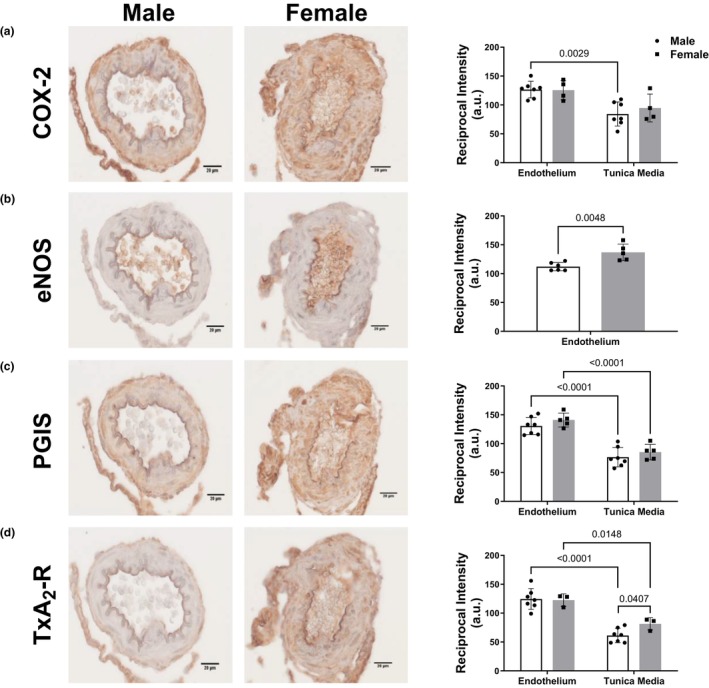
Representative images of immunohistochemical staining and the analysis of COX‐2 (Panel a), eNOS (Panel b), PG synthase (Panel c) and TxA_2_‐R (Panel d) in the endothelial and vascular smooth muscle cell layer of the left MCA in adult male versus female SD rats. Data are mean ± SD; *n* = 3–7.

The differences between male and female groups were compared using unpaired *t*‐test. The immunostaining for various proteins (Figure 2) was analyzed using two‐way analysis of variance (ANOVA) followed by the Tukey post hoc tests (GraphPad Software Inc., La Jolla, CA). All data were presented as mean ± SD. Data analysis for vascular experiments was performed using GraphPad. Individual experimental data from concentration‐response curves for ACh and U‐46619 were fitted to the following logistic curve to determine the maximal response and sensitivity Y = bottom + (top‐bottom)/ (1 + 10(LogEC_50_‐X)*Hill Slope) where X is the logarithm of the concentration and Y is the response. Basal resting tone and active tone (plus 75 mM K+) were expressed as arterial wall tension (AWT) (AWT = force/ 2 × length of vessel) (Pulgar et al., 2014, 2015, 2019). Response to ACh was expressed as % of pre‐constriction and response to U‐46619 was expressed as % of K_MAX_ (maximal response to KCl 75 mM). Sensitivity was expressed as pD2 (pD_2_ = −log [EC_50_]). Data are expressed as mean ± SEM. Values of maximum response and sensitivity were compared by Student's *t*‐test. For all experiments, a *p*‐value less than 0.05 was considered statistically significantly different.

## 3 RESULTS

### 3.1 Physiological characteristics of 25‐week‐old male versus female SD rats

Table 1 shows that male SD rats had greater body weight compared with female SD rats. Females had lower heart and mean kidney weight (normalized to tibia length). There was no difference in systolic or diastolic blood pressures between sexes. Males had a greater pulse pressure versus females. However, female SD rats had a greater heart rate versus male SD.

**TABLE 1:** Physiological characteristics of 25‐week‐old male versus female SD rats.

	Male	Female
Body Weight, g	619.9 ± 99.11	349.0 ± 45.30*
Brain Weight/Tibia Length, g/cm	0.4679 ± 0.04	0.4968 ± 0.02
Heart Weight/Tibia Length, g/cm	0.3080 ± 0.03	0.2394 ± 0.03*
Mean Kidney Weight/Tibia Length, g/cm	0.3736 ± 0.04	0.2708 ± 0.02*
Systolic BP (mmHg)	136.2 ± 11.38	128.6 ± 8.29
Diastolic BP (mmHg)	94.2 ± 9.52	94.7 ± 7.63
Pulse Pressure (mmHg)	42 ± 3.44	33.9 ± 3.98*
Heart Rate (bpm)	343.1 ± 26.46	438.2 ± 30.18*

*Note*: Data are mean ± SD. Comparisons made versus Male by unpaired Student's *t*‐test.

Abbreviations: Bpm, beats per minute; cm, centimeter; g, gram.

* *p* < 0.05 versus male, *n* = 7–11.

### 3.2 Sex differences in left MCA pulsatility index in adult SD rats

**FIGURE 3:**
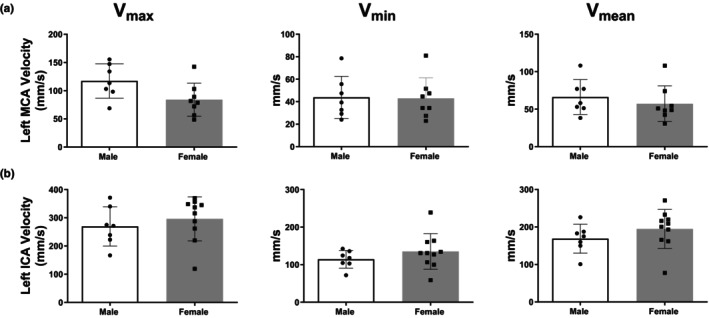
Cerebral blood flow velocities of left MCA and ICA in adult Sprague–Dawley (SD) rats. Left MCA (Panel a) and ICA (Panel b) V_max_, V_min_, and V_mean_ in adult male versus female SD rats. Data are mean ± SD, *n* = 7–10.

Figure 1a,b show color Doppler signal for left ICA and MCA with a typical wave form of MCA. Figure 1c demonstrates that females had lower LMCA PI compared to males. There was no difference in LICA PI between female and male SD (Figure 1d). Additionally, there was no difference in blood flow velocity measurements (V_max_, V_min_, and V_mean_) between sexes for either LMCA or LICA (Figure 3a,b).

### 3.3 No sex differences in systemic arterial stiffness in adult SD rats

**FIGURE 4:**
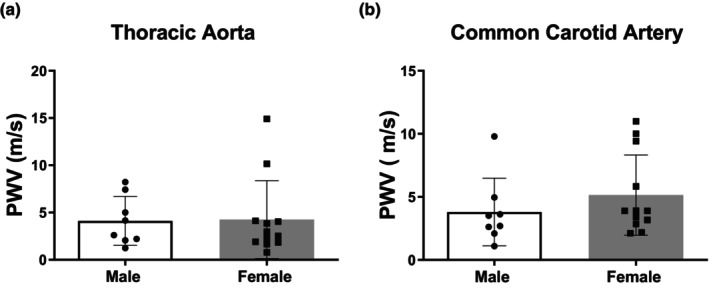
Aortic arch (Panel a) and left common carotid artery (Panel b) pulse wave velocity (PWV) in adult male versus female SD rats. Data are mean ± SD, *n* = 8–12.

There were no differences in thoracic aorta or left common carotid artery PWV between female and male adult SD rats (Figure 4a,b).

### 3.4 Sex differences in the levels of vasoactive targets in MCA

There were no differences in the levels of COX‐2, PGIS, or TxA_2_‐R in the endothelial cell layer of female versus male SD MCA (Figure 2a,c,d). eNOS levels were greater in the endothelial cell layer of LMCA of female versus male SD (Figure 2b). The levels of TxA_2_‐R were greater in the tunica media layer of female versus male SD (Figure 2d). There were no differences in the levels of COX‐2 or PGIS in the tunica media of female versus male SD (Figure 2a,c).

### 3.5 Sex differences in vascular reactivity of isolated MCA segments

**FIGURE 5:**
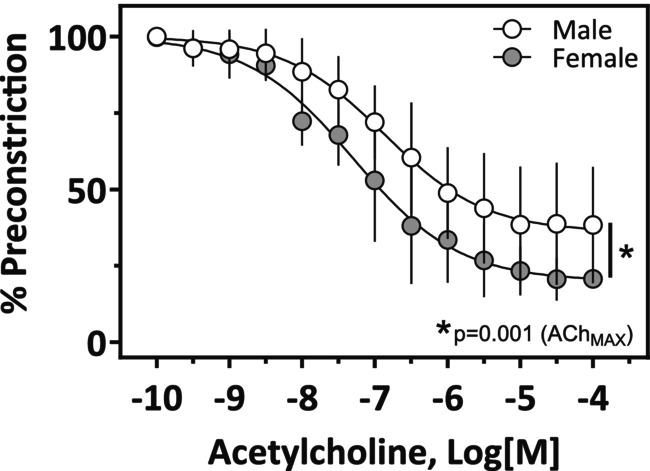
Vasodilatory response to acetylcholine in LMCAs obtained from male and female SD rats. LMCAs from male and female SD were pre‐constricted with U‐46619 (10^−7^ M). Data are mean ± SD, *n* = 5 per group.

**FIGURE 6:**
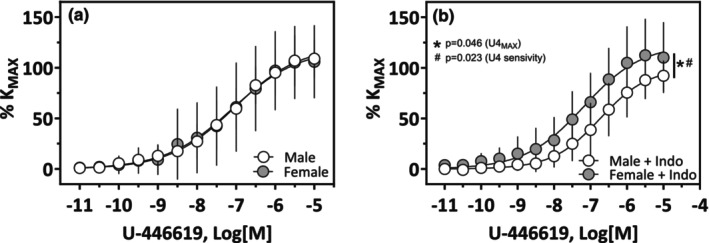
Contraction response of LMCAs to U‐46619 in male and female SD rats. LMCAs from male (*n* = 10) and female (*n* = 13) were exposed to U‐46619 as indicated. Parallel experiments in intact arteries (a) and arteries preincubated with indomethacin (b; 10^−5^ M) are shown. Data are mean ± SD.

LMCAs from male and female SD rats showed similar optimal diameters and basal tone, with a lower active tone observed in female LMCA (Table 2). The vasodilatory response to acetylcholine was greater in female LMCA compared to male LMCA (Figure 5; Table 3). The response to the TxA_2_ agonist U‐46619 was not different between male and female LMCA (Figure 6a), however, in the presence of Indomethacin (10^−5^ M) male LMCA showed a lower maximal response and sensitivity compared to female LMCA (Figure 6b, Table 3).

**TABLE 2:** Structural and functional characteristics of LMCA in 25‐week‐old male versus female SD rats.

	Male	Female
Optimal Diameter, μm	222 ± 19	216 ± 12
Basal Tone, mN/mm	0.6 ± 0.28	0.53 ± 0.23
Active Tone, mN/mm	0.93 ± 0.47	0.61 ± 0.28*

*Note*: Data are mean ± SD. Comparisons made versus Male by unpaired Student's *t*‐test.

* *p* < 0.05 versus Male, *n* = 10–14.

**TABLE 3:** Responses to acetylcholine (ACh) and U‐46619 (U4) in LMCA in 25‐week‐old male versus female SD rats.

	Male		Female	
ACh_MAX_, %pre‐constriction	39 ± 19		81 ± 6*	
ACh sensitivity, pD_2_	7.09 ± 0.27		7.29 ± 0.4	
	Control	+ Indo	Control	+ Indo
U4_MAX_, %K_MAX_	116 ± 20	97 ± 15^#^	109 ± 9	119 ± 34^
U4 sensitivity, pD_2_	7.21 ± 0.5	6.78 ± 0.37^#^	7.28 ± 0.56	7.28 ± 0.8^

*Note*: Data are mean ± SD, *p < 0.05 versus Male; ^#^ *p* < 0.05 versus Control Male; ^^^ *p* < 0.05 versus Control Female; *n* = 5 for acetylcholine (ACh) responses and *n* = 10–13 for U‐46619 (U4) responses. Comparisons made versus Male by unpaired Student's *t*‐test.

## 4 DISCUSSION

The mechanisms underlying sex differences in cerebrovascular function remain unclear, while the impaired MCA reactivity may suggest an increased susceptibility to future cerebrovascular disease. In the present study, we used TCD ultrasound to evaluate LMCA resistance in young adult SD rats. We focused on MCA because it is a major artery supplying blood to a significant portion of the brain. In addition, left MCA is more commonly pathologically affected in young adults (Naess et al., 2006). PI is considered a reliable marker of arterial resistance with increased PI indicating higher vascular resistance (Cho et al., 1997; Kassab et al., 2007; Wielicka et al., 2020). Although there are few studies directly comparing changes in the PI in male versus female MCA, our findings are consistent with previous work demonstrating that young women have decreased MCA PI compared to age‐matched men (Alwatban et al., 2021). Future studies focused on measuring brain oxygenation or cerebrovascular reactivity could be helpful in establishing the hemodynamic consequences of changes in MCA resistance in young adult SD rats.

Large elastic arteries (including aorta and carotid arteries) serve to dampen pulsatile blood flow generated by the heart into a continuous flow. Given its need for steady flow and a relatively narrow range of acceptable perfusion pressures, the brain is particularly affected by the pulsatile blood flow (Mitchell, 2018). Although we have not studied older rats in this study, stiffening of the large elastic arteries that occurs with age may contribute to the increase in cerebral pulsatility that can potentially lead to cerebrovascular dysfunction and cognitive impairment (Reeve et al., 2024). Pulse pressure and PWV increase with age and serve as predictors of cardiovascular disease including stroke and vascular dementia (Mitchell et al., 2008; Waldstein et al., 2008). Compared with men, women experience accelerated vessel stiffening and pulsatile flow with aging. Our results are consistent with human studies where young women have lower PP compared to men (Lefferts et al., 2020; Smulyan et al., 2001). Additionally, we found no sex difference in PWV of the thoracic aorta or common carotid artery consistent with previous studies evaluating abdominal aortic PWV in Dahl salt‐resistant rats (Decano et al., 2016) or aortic arch PWV in glucose‐fed or fructose‐ and high salt‐treated SD rats (Komnenov & Rossi, 2023). It is important to note that there are several different methods to calculate PWV. Similar to our study, Smulynan et al., found no sex differences in aortas of young men and women using the transit‐time method, while Lefferts and colleagues used the local, one‐point technique demonstrating reduced common carotid artery PWV in women compared with men prior to‐ but not after the menopause (Lefferts et al., 2020; Smulyan et al., 2001). Thus, the vascular bed and the technique used to determine PWV need to be considered when making comparisons between different studies.

Regulation of cerebrovascular tone and blood flow is a complex process involving a number of mechanisms including: myogenic responses of smooth muscle to changes in arterial pressure, flow‐metabolism coupling, autonomic nervous system input, vasoactive arachidonic acid (AA) metabolites, and endothelial factors such as eNOS and endothelium‐derived hyperpolarizing factor (EDHF) (Andresen et al., 2006; Cipolla, 2009; Koep et al., 2022; Peterson et al., 2011; You et al., 2005). We show that female SD exhibit greater eNOS staining intensity in the endothelial layer of LMCA sections compared with males. Given that NO is a powerful vasodilator, sex differences in MCA resistance in our study are likely related to local eNOS expression. Previous work demonstrates that estrogen has a dramatic influence on vascular function through modulation of eNOS (Chambliss & Shaul, 2002). Human endothelial cells have intrinsic sexual dimorphism with females demonstrating higher eNOS expression and enzymatic activity (Cattaneo et al., 2017). Estrogen increases eNOS enzymatic activity and levels in an estrogen‐receptor dependent fashion (Hayashi et al., 1995; MacRitchie et al., 1997; Sumi et al., 2001; Sumi & Ignarro, 2003). Additionally, PI in ICA increases after menopause while transdermal estrogen application reduces PI in ICA of postmenopausal women (Gangar et al., 1991). It should be noted that we did not monitor the estrous cycle of rats which reflects the bioavailability of female sex hormones; however, the uterine weight‐to‐tibia length ratio was similar among female rats (0.1399 ± 0. 02 g/cm; 18.69% coefficient of variation). Other hormones, including testosterone and progesterone, also play important roles in vascular function. Progestins counteract the effect of estrogen on PI in carotid arteries (Luckas et al., 1998). Chronic exposure to testosterone may decrease vasodilation and increase vascular tone in castrated male rats (Robison et al., 2019). In addition, the influence of sex hormones on other mechanisms (e.g., autonomic nervous system and flow‐metabolism coupling) may also contribute to sex differences in the regulation of cerebrovascular hemodynamics.

Many AA metabolites (e.g., prostaglandins and thromboxanes) are vasoactive with either vasoconstrictor or vasodilator effects (Bogatcheva et al., 2005). Isoforms of the cyclooxygenase (COX) enzyme are primarily responsible for the initial metabolism of AA; subsequent processing via thromboxane synthase or PGIS produces vasoactive metabolites including thromboxane A_2_ (TxA_2_) or prostacyclin (PGI_2_). We found no significant difference between sexes in the expression of COX‐2. Additionally, there were no differences between sexes in the expression of PGIS, the enzyme responsible for producing the powerful vasodilator PGI_2_. However, the levels of TxA_2_R were greater in the VSMC layer of female SD. Considering that female rats exhibit less VSMCs in the MCA, it is possible the total number of receptors is similar between male and female SD (Wang et al., 2020). Although, we also did not measure the levels of thromboxane synthase, the enzyme responsible for producing TxA_2_, it is possible that the overall availability of TxA_2_ may differ between sexes due to differential expression of thromboxane synthase. In contrast to endothelial cells, greater TxA_2_R levels in VSMC of females MCA does not appear to influence COX‐2 expression. Although the products of AA metabolism are thought to play a somewhat minor role in maintaining cerebrovascular tone (Andresen et al., 2006; Cipolla, 2009; You et al., 2005), the physiologic significance of greater TxA_2_R in the VSMC layer of female MCA in relation to female sex hormone status needs further investigation (Ospina et al., 2003).

Our data also showed differences in local vascular responses between isolated male and female LMCAs. The lower active tone in female LMCAs may reflect their previously reported lower smooth muscle content (Wang et al., 2020), whereas the greater expression of eNOS may explain the increased vasodilatory response we observed in female LMCAs in our study. This increased vasodilatory response may counteract greater levels of the TXA_2_ receptor and be responsible for the similar responses to U‐46619 we observed in male and female LMCAs. Pre‐incubation with the unspecific blocker of the prostanoid pathway indomethacin should decrease the levels of all vasoactive products of AA metabolism, and in this scenario, the greater levels of the TXA_2_ receptor in female LMCAs would also be responsible for the lower U‐46619‐dependent contraction in male but not in female LMCAs.

## 5 CONCLUSION

Overall, we demonstrated sex differences in MCA reactivity and hemodynamics independent from changes in systemic arterial stiffening in young adult SD rats potentially indicating diverse mechanisms underlying vascular resistance in cerebral versus systemic arterial beds. Our data also suggest a potentially protective profile of MCA hemodynamics and reactivity in female compared to male young adult SD rats.

## AUTHOR CONTRIBUTIONS

JWR conceived and designed research, performed experiments, analyzed data, interpreted results of experiments, prepared figures, drafted manuscript, and edited and revised manuscript. XS performed experiments, analyzed data, interpreted results of experiments, edited and revised manuscript. NCD performed experiments, analyzed data, interpreted results of experiments. VMP conceived and designed research, performed experiments, analyzed data, interpreted results of experiments, prepared figures, drafted manuscript, and edited and revised manuscript, and approved final version of manuscript. LMY conceived and designed research, analyzed data, interpreted results of experiments, prepared figures, edited and revised manuscript, and approved final version of manuscript.

## FUNDING INFORMATION

These studies were supported by the National Institutes of Health, Grant/Award Number: R01HL155420, 1R21HD114073‐01 (to LM Yamaleyeva), and in part by the R. Odell Farley Research Fund.

## CONFLICT OF INTEREST STATEMENT

No conflicts of interest, financial, or otherwise are declared by the authors.

## ETHICS STATEMENT

All animal experimental procedures performed in this study were approved by the Institutional Animal Care and Use Committee of the Wake Forest University School of Medicine, Winston‐Salem NC (A21‐057).

## Data Availability

The data are available from the corresponding authors upon reasonable request.
